# Supplementary material for: Predominance of cis-regulatory changes in parallel expression divergence of sticklebacks
Source: eLife. 2019 May 15;8:e43785. doi: 10.7554/eLife.43785 (PMC6550882; doi:10.7554/eLife.43785)
Supplement: Supplementary file 1. [file elife-43785-supp1.docx]

| **Supplementary file 1. Sampling locations** | | |
| --- | --- | --- |
| Sampling locations | Freshwater sampling coordinates | Marine sampling coordinates |
| River Tyne | 55°56'34.8"N 2°47'6"W | 56°0'22.63"N 2°36'47.41"W |
| River Forss | 58°34'22.949"N 3°38'27.916"W | 58°36'32.335"N 3°40'42.404"W |
| River Shiel | 56°44'52.8"N 5°41'52.8"W | 56°46'45.99"N 5°49'44.47"W |
| Little Campbell River | 49°0'43.65"N 122°37'30.44"W | 49°0'58.03"N 122°46'46.37"W |
