# Supplementary material for: Predominance of cis-regulatory changes in parallel expression divergence of sticklebacks
Source: eLife. 2019 May 15;8:e43785. doi: 10.7554/eLife.43785 (PMC6550882; doi:10.7554/eLife.43785)
Supplement: Supplementary file 2. [file elife-43785-supp2.docx]

| **Supplementary file 2. RNA-seq library sequencing and yield.** | | | | | |
| --- | --- | --- | --- | --- | --- |
| **Sample ID** | **Sampling location** | **Ecotype** | **Sequencing run** | **Sequencing lane** | **Mapped reads *** |
| c363_P_FC18_M | Little Campbell River | Freshwater (parent) | 48 | 1 | 41570029 |
| c363_P_FC08_F | Little Campbell River | Marine (parent) | 48 | 1 | 77071816 |
| c358_P_FC12_F | Little Campbell River | Freshwater | 48 | 2 | 51196050 |
| c358_P_FC09_M | Little Campbell River | Marine | 48 | 2 | 62721395 |
| c357_P_FC14_F | Little Campbell River | Freshwater | 48 | 2 | 51219036 |
| c357_P_FC06_M | Little Campbell River | Marine | 48 | 2 | 49172261 |
| c353_P_FC15_M | Little Campbell River | Freshwater | 48 | 2 | 62929997 |
| c353_P_FC05_F | Little Campbell River | Marine | 48 | 2 | 40600091 |
| c209_P_422_M | River Tyne | Freshwater | 39 | 7 | 57545801 |
| c209_P_341_FM | River Tyne | Marine | 39 | 7 | 65354927 |
| c208_P_531_M | River Tyne | Marine | 39 | 7 | 51827643 |
| c208_P_321_FM | River Tyne | Freshwater | 39 | 7 | 59257432 |
| c172_P_533_M | River Tyne | Freshwater (parent) | 14 | 8 | 37475539 |
| c172_P_533_M | River Tyne | Freshwater (parent) | 14 | 3 | 37561905 |
| c172_P_532_F | River Tyne | Marine (parent) | 48 | 2 | 32355769 |
| c172_P_532_F | River Tyne | Marine (parent) | 14 | 8 | 29421104 |
| c172_P_532_F | River Tyne | Marine (parent) | 14 | 3 | 30491397 |
| c169_P_432_FM | River Tyne | Freshwater | 39 | 7 | 46449279 |
| c169_P_342_M | River Tyne | Marine | 39 | 7 | 60308422 |
| c214_P_524_FM | River Shiel | Marine (parent) | 39 | 6 | 63867976 |
| c214_P_512_M | River Shiel | Freshwater (parent) | 39 | 6 | 51993114 |
| c212_P_551_M | River Forss | Freshwater (parent) | 39 | 5 | 78491454 |
| c212_P_454_FM | River Forss | Marine (parent) | 39 | 5 | 53915757 |
| c363_F1_1_M | Little Campbell River | F1 | 48 | 1 | 51918812 |
| c363_F1_1_F | Little Campbell River | F1 | 48 | 1 | 75548850 |
| c363_F1_2_M | Little Campbell River | F1 | 48 | 1 | 56909054 |
| c363_F1_2_F | Little Campbell River | F1 | 48 | 1 | 49711377 |
| c172_F1_04_M | River Tyne | F1 | 14 | 8 | 43447943 |
| c172_F1_04_F | River Tyne | F1 | 14 | 8 | 30068470 |
| c172_F1_20_M | River Tyne | F1 | 14 | 3 | 26736357 |
| c172_F1_20_F | River Tyne | F1 | 14 | 3 | 42657169 |
| c214_F1_2_M | River Shiel | F1 | 39 | 6 | 67202528 |
| c214_F1_2_FM | River Shiel | F1 | 39 | 6 | 51160598 |
| c214_F1_1_M | River Shiel | F1 | 39 | 6 | 58378310 |
| c214_F1_1_FM | River Shiel | F1 | 39 | 6 | 62005808 |
| c212_F1_2_M | River Forss | F1 | 39 | 5 | 52427751 |
| c212_F1_2_FM | River Forss | F1 | 39 | 5 | 62315657 |
| c212_F1_1_M | River Forss | F1 | 39 | 5 | 56085728 |
| c212_F1_1_FM | River Forss | F1 | 39 | 5 | 53416036 |
| * uniquely mapped reads | | | | | |
