# Supplementary material for: Predominance of cis-regulatory changes in parallel expression divergence of sticklebacks
Source: eLife. 2019 May 15;8:e43785. doi: 10.7554/eLife.43785 (PMC6550882; doi:10.7554/eLife.43785)
Supplement: Supplementary file 4. [file elife-43785-supp4.docx]

| **Supplementary file 4. Criteria for defining divergence classes following (Landry et al., 2005).** | | | | |
| --- | --- | --- | --- | --- |
| **Divergence class** | **Alleles - Binomial test** | **Parents - Binomial test** | **ASE versus Parents - Fisher test** | **Additional** |
| cis | ** (A1≠A2) | ** (P1≠ P2) | NS (P1/P2 = A1/A2) | NA |
| trans | NS (A1= A2) | ** (P1 ≠ P2) | ** (P1/P2 ≠ A1/A2) | NA |
| cis + trans | ** (A1≠A2) | ** (P1≠ P2) | ** (P1/P2 ≠ A1/A2) | log2(P1/P2)/log2(A1/A2) > 1 |
| cis - trans | ** (A1≠A2) | ** (P1≠ P2) | ** (P1/P2 ≠ A1/A2) | log2(P1/P2)/log2(A1/A2) < 1 |
| compensatory | ** (A1≠A2) | NS (P1 = P2) | ** (P1/P2 ≠ A1/A2) | NA |
| conserved | NS (A1 = A2) | NS (P1 = P2) | NS (P1/P2 = A1/A2) | NA |
| A1 - allele 1, A2 - allele 2, P1 - parent 1, P2 - parent 2, ** - statistically significant with FDR 10%, NS - non-significant with FDR 10% | | | | |
