# Supplementary material for: Predominance of cis-regulatory changes in parallel expression divergence of sticklebacks
Source: eLife. 2019 May 15;8:e43785. doi: 10.7554/eLife.43785 (PMC6550882; doi:10.7554/eLife.43785)
Supplement: Supplementary file 5. [file elife-43785-supp5.docx]

| **Supplementary file 5. DNA sequencing yield.** | | | |
| --- | --- | --- | --- |
| **Sample ID** | **Sampling location** | **Ecotype** | **Mapped reads *** |
| FC14 | Little Campbell River | Freshwater | 17438204 |
| FC13 | Little Campbell River | Freshwater | 23171229 |
| FC12 | Little Campbell River | Freshwater | 25146843 |
| FC16 | Little Campbell River | Freshwater | 30005091 |
| FC18 | Little Campbell River | Freshwater | 38399784 |
| FC15 | Little Campbell River | Freshwater | 15152985 |
| c363_P_FC08_F | Little Campbell River | Freshwater (parent) | 58346540 |
| LITC_DWN_4 | Little Campbell River | Marine | 28238410 |
| LITC_DWN_5_F | Little Campbell River | Marine | 37997779 |
| LITC_DWN_6_F | Little Campbell River | Marine | 46675891 |
| LITC_DWN_7_F | Little Campbell River | Marine | 27626027 |
| LITC_DWN_8_F | Little Campbell River | Marine | 30613683 |
| LITC_DWN_9_F | Little Campbell River | Marine | 25885479 |
| c363_P_FC18_M | Little Campbell River | Marine (parent) | 36326200 |
| Tyne8_27 | River Tyne | Freshwater | 57821343 |
| Tyne8_4 | River Tyne | Freshwater | 75419343 |
| Tyne8_7 | River Tyne | Freshwater | 80784624 |
| Tank422_4 | River Tyne | Freshwater | 120054557 |
| Tyne8_2 | River Tyne | Freshwater | 89618061 |
| Tyne8_1 | River Tyne | Freshwater | 58244138 |
| c172_P_533_M | River Tyne | Freshwater (parent) | 56617456 |
| Tyne2_16\|2015 | River Tyne | Marine | 89914271 |
| Tyne2_18 | River Tyne | Marine | 28775921 |
| Tyne2_20 | River Tyne | Marine | 55538908 |
| Tyne2_12 | River Tyne | Marine | 15830364 |
| Tyne2_16\|2014 | River Tyne | Marine | 24148885 |
| Tyne2_14 | River Tyne | Marine | 108126294 |
| c172_P_532_F | River Tyne | Marine (parent) | 66347185 |
| c214_P_512_M | River Shiel | Freshwater (parent) | 57188122 |
| c214_P_524_F | River Shiel | Marine (parent) | 61037784 |
| c212_P_551_M | River Forss | Freshwater (parent) | 53922480 |
| c212_P_454_F | River Forss | Marine (parent) | 27969046 |
| * samtools view -F 0x4 *.bam \| cut -f 1 \| sort \| uniq \| wc -l | | | |
